# Glutathione S-Transferase P Influences Redox and Migration Pathways in Bone Marrow

**DOI:** 10.1371/journal.pone.0107478

**Published:** 2014-09-12

**Authors:** Jie Zhang, Zhi-Wei Ye, Peng Gao, Leticia Reyes, Elizabeth E. Jones, Melissa Branham-O’Connor, Joe B. Blumer, Richard R. Drake, Yefim Manevich, Danyelle M. Townsend, Kenneth D. Tew

**Affiliations:** 1 Department of Cell and Molecular Pharmacology and Experimental Therapeutics, Medical University of South Carolina, Charleston, South Carolina, United States of America; 2 Department of Pharmaceutical and Biomedical Sciences, Medical University of South Carolina, Charleston, South Carolina, United States of America; Witten/Herdecke University, Germany

## Abstract

To interrogate why redox homeostasis and glutathione S-transferase P (GSTP) are important in regulating bone marrow cell proliferation and migration, we isolated crude bone marrow, lineage negative and bone marrow derived-dendritic cells (BMDDCs) from both wild type (WT) and knockout (*Gstp1/p2* ^−/−^) mice. Comparison of the two strains showed distinct thiol expression patterns. WT had higher baseline and reactive oxygen species-induced levels of S-glutathionylated proteins, some of which (sarco-endoplasmic reticulum Ca^2+^-ATPase) regulate Ca^2+^ fluxes and subsequently influence proliferation and migration. Redox status is also a crucial determinant in the regulation of the chemokine system. CXCL12 chemotactic response was stronger in WT cells, with commensurate alterations in plasma membrane polarization/permeability and intracellular calcium fluxes; activities of the downstream kinases, ERK and Akt were also higher in WT. In addition, expression levels of the chemokine receptor CXCR4 and its associated phosphatase, SHP-2, were higher in WT. Inhibition of CXCR4 or SHP2 decreased the extent of CXCL12-induced migration in WT BMDDCs. The differential surface densities of CXCR4, SHP-2 and inositol trisphosphate receptor in WT and *Gstp1/p2* ^−/−^ cells correlated with the differential CXCR4 functional activities, as measured by the extent of chemokine-induced directional migration and differences in intracellular signaling. These observed differences contribute to our understanding of how genetic ablation of GSTP causes higher levels of myeloproliferation and migration.

## Introduction

The bone marrow produces all the differentiated hematopoietic cells for peripheral blood. This tissue is extremely sensitive to alterations in redox homeostasis, as proliferation and differentiation are influenced by physiological changes in a number of factors that are sensitive to reactive oxygen species (ROS; [1], [2]). ROS and the oxidation/reduction of thiols have key roles in cell signaling events that regulate a variety of biological functions [3]–[7]. Reduced glutathione (GSH) is the most abundant non-protein thiol at cellular concentrations that range from 0.1 to 10 mM [8]. Glutathione S-transferase P (GSTP) is one of a family of GST isozymes and has functions as a catalytic enzyme, protein chaperone, kinase regulator and in regulating the forward reaction of protein S-glutathionylation [1], [9]. This post-translational modification occurs in certain protein clusters that have roles in events regulating cell proliferation [10]. It is reasonable to speculate that the difference in ROS levels in myeloid progenitor and quiescent hematopoietic stem cells (HSCs) may act in intracellular signaling events that drive HSC differentiation and that modulation of redox-sensitive cysteines through S-glutathionylation may have a key role in these events.

As early as 1953 a role for cysteines and thiols in bone marrow cell proliferation was established [11]. Various drugs that disrupt thiol homeostasis have been shown to exert a redox-based influence on components of bone marrow proliferation. For example, N-acetyl cysteine has been used in the management of patients with HIV, enabling more robust immune responses through T helper cells [12]; NOV-002 (a glutathione disulfide mimetic) enhances marrow recovery following immunosuppressive drugs in both rodents [13] and humans [14]; TLK199, [γ-glutamyl-*S*-(benzyl)-cysteinyl-*R*-(-) phenyl glycine diethyl ester] (*Telintra*) as a GSH peptidomimetic inhibitor of GSTP stimulates myeloproliferation in both rodents [15] and man [16], [17]. In mice, *Telintra* increases all peripheral blood cell lineages in wild type mice as compared to GSTP-deficient mice. Even in the absence of *Telintra*, GSTP-null animals have increased hematopoietic progenitor cell (HPC) numbers, myeloid cell differentiation and proliferation [18]. *Telintra* has shown positive results (decreased requirements for red blood cell, platelet and growth factor support) in ongoing Phase 2 clinical trials for patients with myelodysplastic syndrome [16], [17], a stem cell disorder characterized by ineffective blood cell production and an increased risk for transformation to acute leukemia.

Bone marrow is a relatively hypoxic tissue (1% to 2% O_2_) [19], but within the three-dimensional marrow compartment, self-renewing HSCs, HPCs and mature blood cells are able to migrate in a site/niche-specific fashion regulated by factors such as O_2_ and Ca^2+^ gradients. At any given time, approximately 75% of HSCs are in a quiescent phase of the cell cycle [20]. At the bone-bone marrow interface (osteoblastic niche), the microenvironment favors HSC quiescence, while closer to blood vessels (vascular niche), proliferation and differentiation is more likely [21]–[25]. Osteoclast and osteoblast-mediated bone remodeling results in an increased extracellular Ca^2+^ in the endosteum and Ca^2+^ gradient between osteoblastic and vascular niches, enabling HSCs to sense and migrate appropriately [26]. Adhesive molecules, cytokines and chemokine signaling determine population and niche characteristics. The chemokine CXCL12 plays an essential role in retaining and maintaining HSCs in bone marrow and depletion of a related cytokine, CXCR4, increases HSCs in the peripheral blood [27], [28]. The interplay between ROS and thiol balance/gradients is critical to myeloproliferation and/or migration, as the redox status can be regulated by shifts of thiol-disulfide equilibrium [2]. Since pharmaceutical inhibition of GSTP has translational applications in myeloproliferation, the present studies were designed to address how genetic ablation of GSTP impacts bone marrow cell redox parameters and influences downstream events that contribute to proliferation and migration in this tissue.

## Results

### Increased DNA synthesis in *Gstp1/p2* ^−/−^ bone marrow cell populations

**Figure 1:**
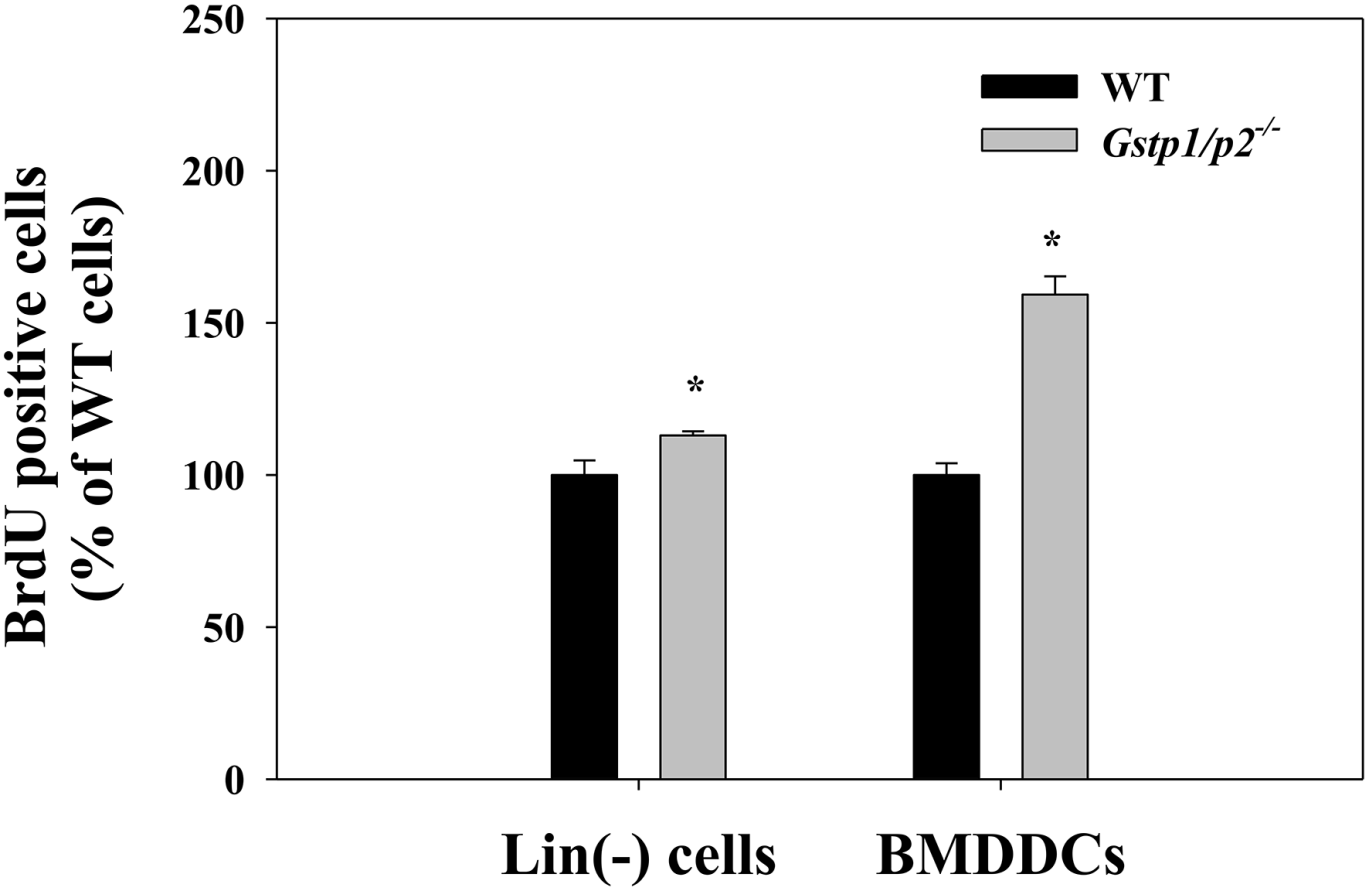
Increased cell proliferation in *Gstp1/p2* ^−/−^ bone marrow cell populations. Wild type or *Gstp1/p2* ^−/−^ Lin(−) cells and BMDDCs were seeded at 0.2×10^6^ cells/ml in Lin(−) cell medium or DC medium and incubated for 48 h. BrdU solution was then added for an extra 4 h. The incorporation of BrdU into DNA was measured with monoclonal anti-BrdU antibodies in an ELISA format. Values are means (*±SD*) from three independent experiments, with asterisks (*) indicating statistical significant differences between WT and *Gstp1/p2* ^−/−^ Lin(−) cells or BMDDCs (*p*<0.05).

Cell proliferation was assessed by BrdU incorporation. In the presence of stem/progenitor cell growth factors (stem cell factor, thrombopoietin and Flt3L), BrdU incorporation was ∼10% higher in the knockout (*Gstp1/p2* ^−/−^) lineage-negative (Lin(−)) cells, compared to wild type (WT) Lin(−) cells. *Gstp1/p2* ^−/−^ bone marrow derived-dendritic cells (BMDDCs) had a 65% higher DNA synthesis rate than WT cells in response to GM-CSF stimulation. For both Lin(−) cells and BMDDCs, significant differences were observed between WT and *Gstp1/p2* ^−/−^ mice (Fig. 1). These results support our previous publication that ablation of GSTP either genetically or pharmacologically results in the over-production of lymphoid, erythroid and myeloid lineage cell lineages as well as platelets [26].

### Altered redox status in *Gstp1/p2* ^−/−^ bone marrow cell populations

**Figure 2:**
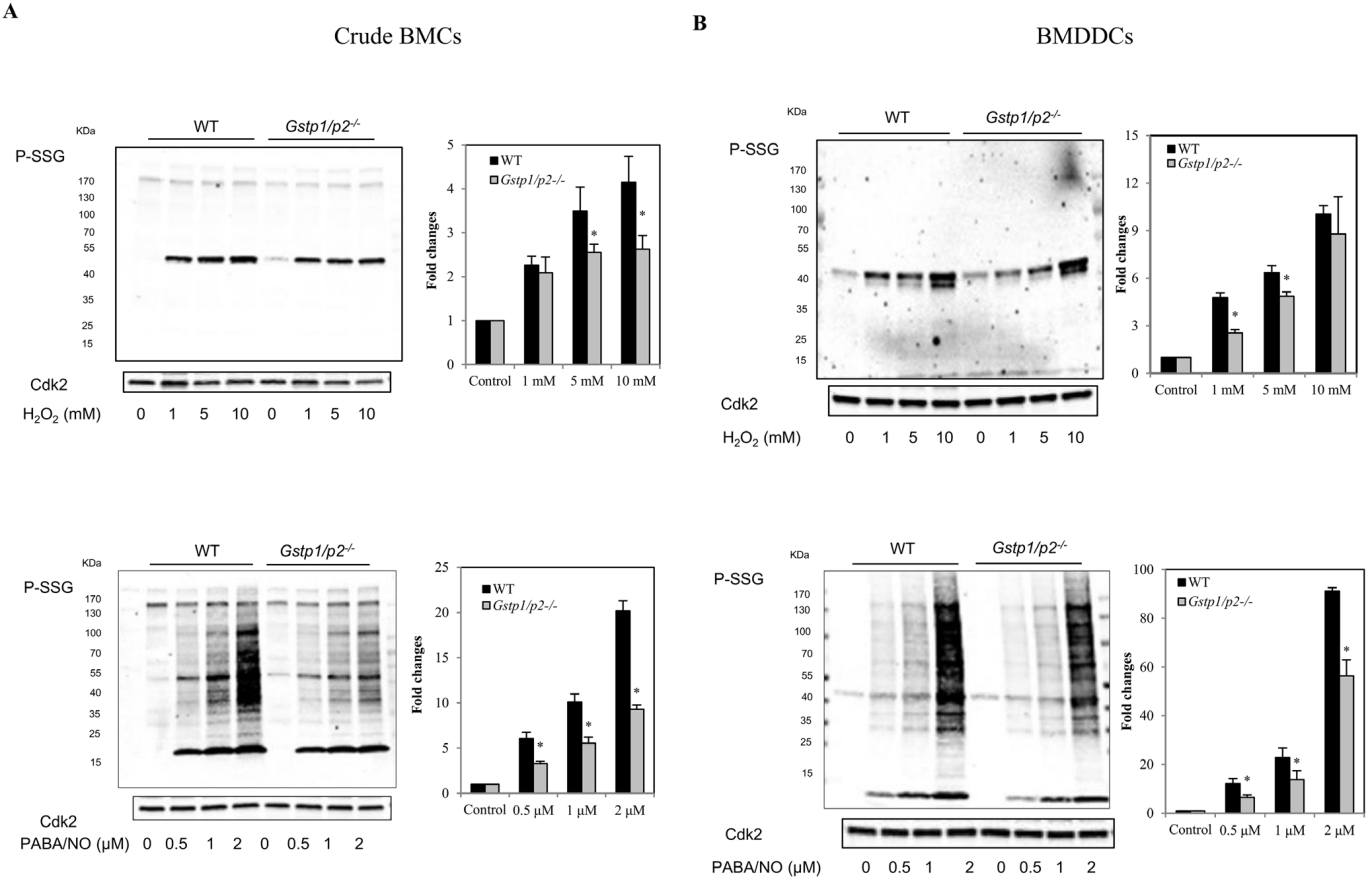
Effect of GSTP ablation on protein S-glutathionylation responses to ROS or RNS. (A) Crude BMCs and (B) BMDDCs derived from WT or *Gstp1/p2* ^−/−^ mice were exposed to various concentrations of H_2_O_2_ (1, 5 or 10 mM) (left panel) or PABA/NO (0.5, 1 or 2 µM) (right panel), at 37°C for 15 min. Proteins were separated by non-reducing SDS-PAGE and protein S-glutathionylation evaluated by immunoblot with mouse anti-GSH antibodies. Even loading of protein was confirmed by probing with rabbit anti-Cdk2 antibodies. Goat anti-mouse and goat anti-rabbit fluorescent secondary antibodies were used and immunoblots imaged with a two-channel IR fluorescent Odyssey CLx imaging system. S-glutathionylation levels were quantified and normalized relative to Cdk2 loading for each concentration point. The ratio from no drug treatment was assigned a value of one and all other ratios are shown relative to this value. Bars represent the means (*±SD*) from three independent experiments.

Crude bone marrow cells (BMCs) or BMDDCs derived from WT or *Gstp1/p2* ^−/−^ mice were used to evaluate the dynamics of S-glutathionylation following exposure to reagents that induce either oxidative (H_2_O_2_) or nitrosative stress (PABA/NO; (O^2^-{2,4-dinitro-5-[4-(N-methylamino)benzoyloxy]phenyl}1-(N,N-dimethylamino)diazen-1-ium-1,2-diolate), a diazeniumdiolate prodrug which releases NO [29]). PABA/NO-induces limited levels of protein nitrosylation/nitration and high levels of S-glutathionylation [30], [31]. As shown in Fig. 2, monoclonal anti-GSH antibodies directed against the GS-moiety detected S-glutathionylated proteins in BMCs and BMDDCs following either H_2_O_2_ or PABA/NO treatment. Under basal conditions, low levels of S-glutathionylated proteins were detectable in BMCs or BMDDCs (primarily actin [32]). H_2_O_2_ treatment produced S-glutathionylation of a limited number of proteins, while PABA/NO led to rapid S-glutathionylation of numerous proteins. A dose-dependent increase in total protein S-glutathionylation was observed following either H_2_O_2_ or PABA/NO in both BMCs and BMDDCs. Following drug treatments, S-glutathionylation levels of proteins in *Gstp1/p2* ^−/−^ cells were consistently lower than WT.

**Figure 3:**
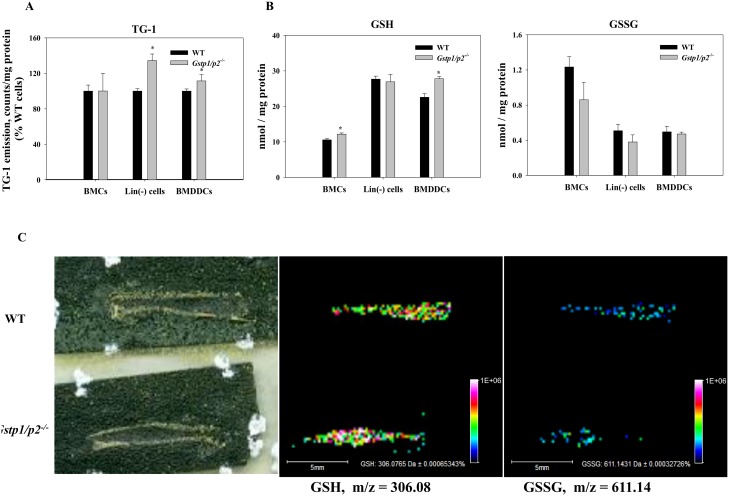
Protein thiols, GSH and GSSG levles in bone marrow cells. (*A, B)* Intracellular reduced protein thiols (A), and GSH/GSSG levels (B) in crude BMCs, Lin(−) cells and BMDDCs. Intracellular reduced thiol and GSH levels were measured by means of a sulfhydryl-specific fluorescent probe; intracellular GSSG levels were determined based on the reduction of GSSG in the presence of glutathione reductase and NADPH and on measurement of NADPH fluorescence decrease. Values are means (±*SD*) from at least three independent experiments, with asterisks (*) indicating statistical significant differences between (*p*<0.05). (*C)* Representative MALDI-MS images of GSH and GSSG in sectioned femur showing bone marrow distribution in WT and *Gstp1/p2* ^−/−^ mice. From left to right: scanned image of matrix sprayed MALDI slide of mouse femur with bone marrow; corresponding images of: GSH ions at m/z = 306.08 and GSSG ions at m/z = 611.14. Color heat map of the data points in the GSH and GSSG images represent averaged individual ion signal intensities of the spots.

The impact of GSTP expression on the redox state of the intracellular protein thiol levels was further examined (Fig. 3A). Significantly higher levels of reduced intracellular protein thiols were observed in *Gstp1/p2* ^−/−^ Lin(−) cells and BMDDCs when compared to WT cells.

In addition, cysteine reactive Isotope-coded affinity tag (ICAT) labeling and LC/MS specifically identified 333 thiol active proteins, of which we selected proteins where abundance ratios between *Gstp1/p2* ^−/−^ and WT were >1.2, or <0.8, including: vimentin, apoptotic chromatin condensation inducer 1, transitional endoplasmic reticulum ATPase, aldoketo-reductase family 1, peroxiredoxin 4, rho GTPase-activating protein 17, triose-phosphate isomerase 1, ras GTPase-activating-like protein IQGAP1, prolyl 4-hydroxylase, S100 calcium binding protein A9, serine/threonine kinase 16 (Table 1).

**Table 1:** List of selected proteins and their abundance ratios between WT and *Gstp1/p2* ^−/−^ BMDDCs.

Accession	Gene names	Protein names	Abundance ratio(GSTP1/P2^−/−^/WT)
31982755	Vim	Vimentin	1.6
9625006	Acin1	Apoptotic chromatincondensation inducer 1	1.6
94408013	Vcp	Transitional endoplasmicreticulum ATPase	1.4
10946870	Akr1a4	Aldo-keto reductase family 1,member A4 (aldehyde reductase)	1.4
7948999	Prdx4	Peroxiredoxin 4	1.2
169790947	Arhgap17	Rho GTPase-activating protein 17	1.2
6678413	Tpi1	Triose-phosphate isomerase 1	1.2
242332572	Iqgap1	Ras GTPase-activating-likeprotein IQGAP1	1.2
42415475	P4hb	Prolyl 4-hydroxylase, betapolypeptide	1.2
6677837	S100a9	S100 calcium binding proteinA9 (calgranulin B)	0.7
31543784	Stk16	Serine/threonine protein kinase 16	0.6

We also measured the *in situ* levels of reduced and oxidized glutathione (GSH and GSSG) in bone marrow populations derived from WT and *Gstp1/p2* ^−/−^ mice. As shown in Fig. 3B, compared to WT cells, levels of reduced GSH were higher, whereas levels of oxidized GSSG were lower in *Gstp1/p2* ^−/−^ crude BMCs, Lin(−) cells or BMDDCs. Significant differences were only observed in GSH levels in *Gstp1/p2* ^−/−^ crude BMCs and BMDDCs when compared to WT cells. In addition, a MALDI-MS bone imaging method was developed to visualize GSH and GSSG simultaneously in bone marrow tissues without any labeling (Fig. 3C). This method generated a multidimensional spatial expression map of the biomolecules directly from a tissue section. As seen in the differential ion intensities of GSH and GSSG, the bone marrow distribution results were consistent with the biochemical measurements showing that differences in qualitative distribution of GSH and GSSG were present between the WT and *Gstp1/p2* ^−/−^ samples.

### Altered intracellular calcium dynamics in *Gstp1/p2* ^−/−^ bone marrow cell populations

**Figure 4:**
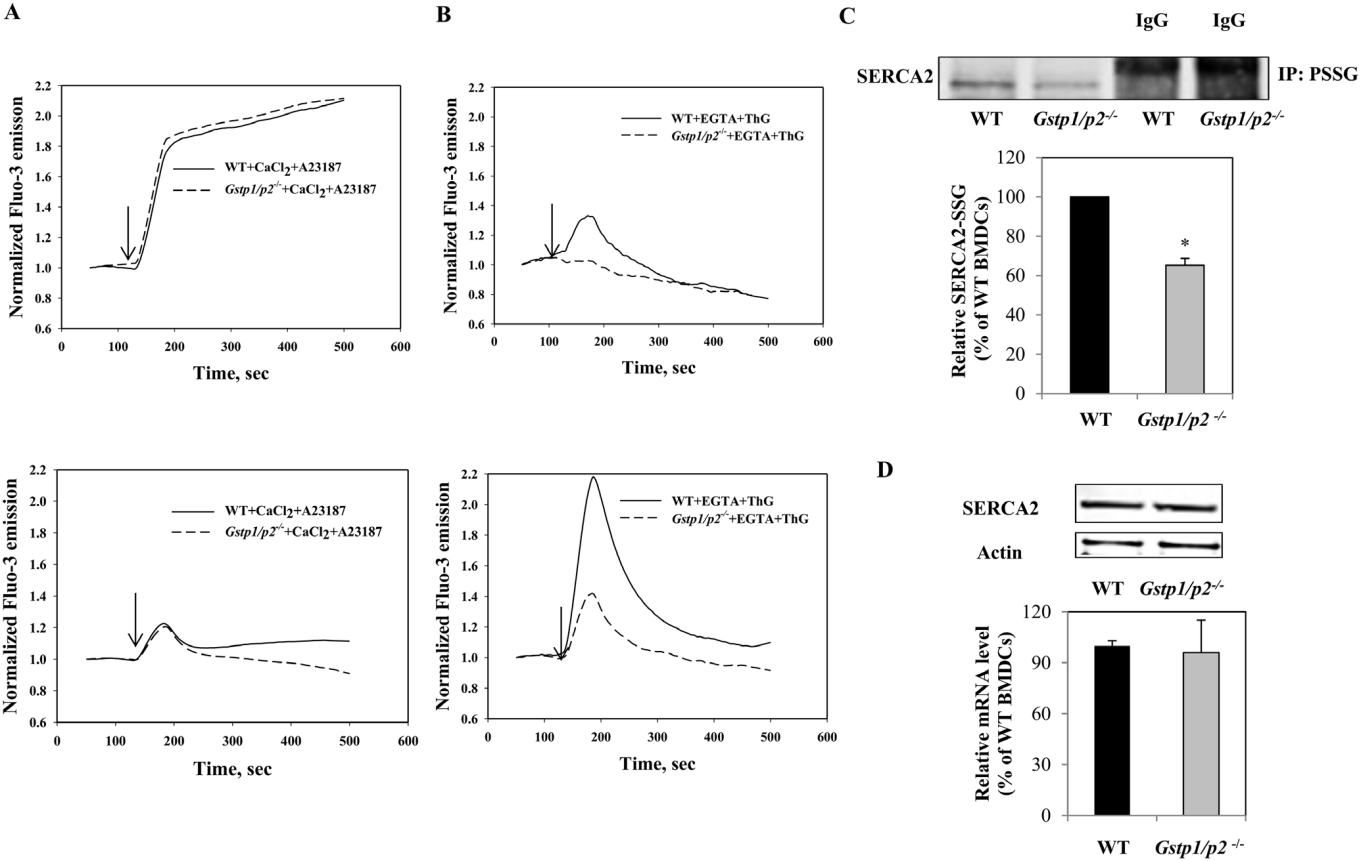
Intracellular calcium dynamics in bone marrow cell populations. Intracellular calcium oscillations in WT or *Gstp1/p2* ^−/−^ Lin(−) cells and BMDDCs with responses to either 1 µM A23187 (*A*), or 1 µM ThG (*B*). Fluo-3-AM-labeled cells were re-suspended in PBS containing 100 µM CaCl_2_ (Ca^2+^-extracellular solution) or 2 mM EGTA (zero Ca^2+^-extracellular solution) immediately prior to use. The kinetics of intracellular calcium changes were measured with a spectrofluorometer (Ex = 506 nm, Em = 526 nm). The arrows indicate the addition of effectors. Data are representative traces of three independent experiments. (*C)* S-glutathionylation of SERCA2 in WT or *Gstp1/p2* ^−/−^ BMDDCs under basal conditions. Five hundred micrograms of protein were immunoprecipitated using mouse anti-GSH antibodies with protein A/G-agarose beads. Samples were analyzed by subsequent non-reducing SDS-PAGE and immunoblots probed using goat anti-SERCA2 antibodies. As a reagent control, cell lysates were incubated with antibody control IgG and subjected to the same procedures. (*D)* SERCA2 basal levels in BMDDCs. Protein levels were evaluated by immunoblotting. Actin served as a loading control. Relative gene expressions were quantified by Real-Time RT-PCR. Bars represent the means *(±SD*) from three independent experiments.

Fig. 4 shows typical calcium responses of Lin(−) cells and BMDDCs after stimulation with either A23187 or Thapsigargin (ThG). A23187 is an ionophore that forms lipid-soluble complexes with divalent metal cations, increasing specific permeability of membranes to Ca^2+^. A23187 induced a rapid increase in [Ca^2+^]_i_, followed by a sustained high level of [Ca^2+^]_i_ in Lin(−) cells. Alternatively, only a transient increase in [Ca^2+^]_i_ was observed in BMDDCs, with no obvious differences between WT and *Gstp1/p2* ^−/−^ cells (Fig. 4A). Inhibition of the sarco-endoplasmic reticulum Ca^2+^-ATPase (SERCA) pump by ThG is a commonly used method for manipulating calcium stores. ThG-induced rises in [Ca^2+^]_i_ reflect the passive leak of Ca^2+^ from the endoplasmic reticulum (ER) following SERCA inhibition [33], [34]. In the absence of extracellular Ca^2+^, ThG induced a rapid and transient elevation of [Ca^2+^]_i_ in both Lin(−) cells and BMDDCs. Interestingly, *Gstp1/p2* ^−/−^ Lin(−) cells and BMDDCs showed significantly lower Ca^2+^ responses to ThG compared to WT cells (Fig. 4B), implying that SERCA activity may be different between WT and *Gstp1/p2* ^−/−^ cells. Supporting this conclusion, S-glutathionylation increases SERCA2 activity [35] and GSTP promotes S-glutathionylation [32]. Therefore, we investigated whether GSTP influenced the S-glutathionylation level of SERCA2 in BMDDCs. To detect S-glutathionylated SERCA2 under control conditions, immunoprecipitation with the anti-GSH antibody followed by immunoblotting with the anti-SERCA2 antibodies was used. As shown in Fig. 4C, compared with *Gstp1/p2* ^−/−^ BMDDCs, WT cells have significant higher levels of S-glutathionylated SERCA2 under control conditions. There were no quantitative differences in SERCA2 mRNA levels or protein expression levels between WT and *Gstp1/p2* ^−/−^ cells (Fig. 4D).

### Altered CXCL12/CXCR4 signaling in *Gstp1/p2* ^−/−^ bone marrow cell populations

**Figure 5:**
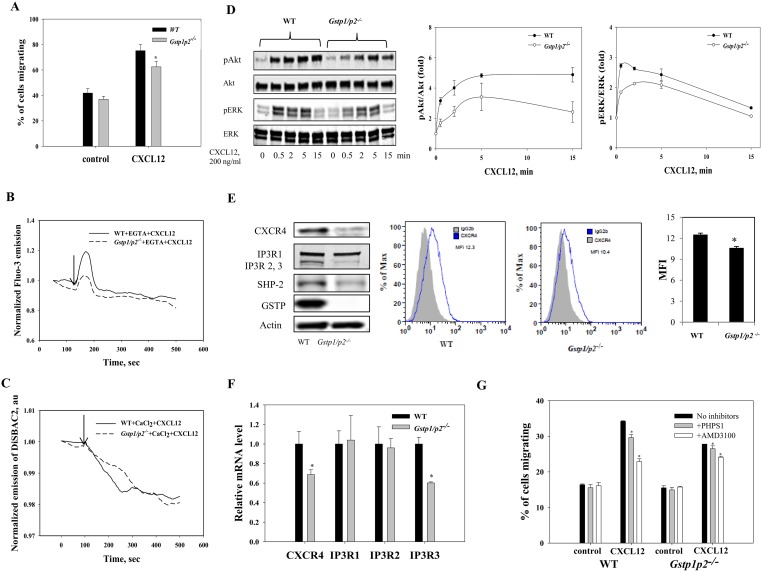
BMDDC cell responses to CXCL12. *(A)* Migration of BMDDCs to CXCL12. Wide type and *Gstp1/p2* ^−/−^ BMDDCs were either left untreated (control) or stimulated with 200 ng/ml CXCL12 for 16 h. Values are average percentages of migration (±*SD*) from six independent experiments with asterisks (*) indicating statistical significant differences between WT and *Gstp1/p2* ^−/−^ BMDDCs (*p*<0.05). (*B, C*) Intracellular calcium (B); plasma membrane potential dynamics (C) in WT and *Gstp1/p2* ^−/−^ BMDDCs in response to CXCL12. Arrows indicate the addition of CXCL12. Data are representative traces of three independent experiments. (*D*) Kinetics of Akt and ERK phosphorylation in BMDDCs with response to CXCL12. Wild type and *Gstp1/p2* ^−/−^ BMDDCs were serum starved for 2 h. 200 ng/ml CXCL12 was then added and cells were collected at various time points. Representative immunoblots show phosphorylation of Akt and ERK in WT and *Gstp1/p2* ^−/−^ BMDDCs after stimulation with CXCL12 for the indicated periods. p-Akt or p-ERK levels were quantified and normalized relative to total Akt or ERK protein for each time point. The ratio (p-Akt:total Akt or p-ERK:total ERK) at 0 min CXCL12 exposure was assigned a value of one and all other ratios are shown relative to this value. Bars represent the means (±*SD*) from three independent experiments. (*E, F*) Impact of GSTP ablation on CXCR4, IP3R and SHP-2 levels in BMDDCs. Total protein levels were evaluated by immunoblots. Actin served as a loading control. Surface expression of CXCR4 was further analyzed by flow cytometry. Representative histograms are shown: CXCR4 expression in WT and *Gstp1/p2* ^−/−^ BMDDCs compared to isotype control showing the mean fluorescence intensity (MFI) and statistical summary of three independent measurements presented as means (±*SD*) with asterisks (*) indicating statistically significant differences between WT and *Gstp1/p2* ^−/−^ BMDDCs (p<0.05). (*E*). Relative gene expression levels were quantified by Real-Time RT-PCR. Bars represent the means *(±SD*) from three independent experiments (*F*). (*G*) Effect of SHP-2 specific inhibitor PHPS1 and CXCR4 specific antagonist AMD3100 on CXCL12-stimulated chemotaxis of BMDDCs. WT and *Gstp1/p2* ^−/−^ BMDDCs were either untreated (control) or pretreated with 10 µM PHPS1 or 1 µM AMD3100 for 2 h, and then cells were either left untreated or stimulated with 200 ng/ml CXCL12 for 6 h. Values are average percentages of specific chemotaxis (percentage of cells migrating to medium alone was subtracted from the percentage of cells migrating to medium with CXCL12) (±*SD*) from three independent experiments, with asterisks (*) indicating statistically significant differences between control and drug treatments (*p*<0.05). Chemotaxis of WT cells in the absence of inhibitor is considered 100% migration.

CXCL12 binds primarily to CXCR4 and initiates divergent signaling pathways downstream of ligand binding, resulting in a number of important cellular responses including chemotaxis, mobilization of intracellular calcium and activation of ERK1/2 and Akt kinases. CXCL12-mediated chemotaxis is mediated at least in part by activation of PI3 kinase/Akt and ERK1/2. Calcium mobilization by CXCL12 is achieved via phospholipase C beta activation and formation of inositol trisphosphate (IP3) and diacylglycerol. IP3/IP3 receptor (IP3R) signaling triggers the opening of the Ca^2+^ channel on the surface of ER, and thus release of Ca^2+^ into the cytoplasm [36], [37]. We compared the results of GSTP ablation in Lin(−) cells (Fig. S1) and BMDDCs (Fig. 5) on chemotaxis, calcium mobilization, plasma membrane potential and intracellular signaling (Akt and ERK activation) after CXCL12 treatment. Both Lin(−) cells and BMDDCs migrated toward CXCL12. The number of Lin(−) cells and BMDDCs that migrated in response to CXCL12 was significantly higher than those not exposed to CXCL12 as a chemoattractant (control). This CXCL12-dependent migration was stronger in WT Lin(−) cells and BMDDCs than in *Gstp1/p2* ^−/− ^cells (Fig. S1A and Fig. 5A).

Figs. S1B and Fig. 5B show the dynamics of intracellular calcium in Lin(−) cells and BMDDCs after stimulation with CXCL12. CXCL12 induced a rapid and transient elevation of [Ca^2+^]_i_ in both Lin(−) cells and BMDDCs through IP3/IP3R signaling. Interestingly, similar to the results obtained with ThG-induced calcium release from ER stores through SERCA inhibition, *Gstp1/p2* ^−/−^ Lin(−) cells and BMDDCs showed significantly different Ca^2+^ responses to CXCL12 as compared to WT cells. Stronger intracellular calcium oscillations were observed in WT Lin(−) cells and BMDDCs than in *Gstp1/p2* ^−/− ^cells following CXCL12. The response in WT cells was characterized by a relatively rapid rate of rise and robust peak change in [Ca^2+^]_i_, whereas the response in *Gstp1/p2* ^−/−^ cells was characterized by a relatively slow rate of rise and small peak change in [Ca^2+^]_i_.

[Ca^2+^]_i_ affects ion permeability and membrane potential and so we determined whether different Ca^2+^ responses between WT and *Gstp1/p2* ^−/− ^cells were also reflected by different potential responses under CXCL12 stimulation. Plasma membrane potential was determined using the slow-response potential-sensitive dye, bis-(1,3-diethylthiobarbituric acid)trimethine oxonol (DiSBAC2(3)). The anionic dye can enter depolarized cells where it binds to intracellular proteins or membranes and exhibits enhanced fluorescence. Increased depolarization results in additional influx of the anionic dye and thus, an increase in fluorescence. Conversely, hyperpolarization is indicated by a decrease in fluorescence. Fig. S1C and Fig. 5C show the dynamics of plasma membrane potential in Lin(−) cells and BMDDCs following stimulation with CXCL12. Following CXCL12 stimulation, depolarization of plasma membranes was detected in both WT and *Gstp1/p2* ^−/−^ Lin(−) cells, whereas delayed, amplified and reversible plasma membrane depolarization was observed in *Gstp1/p2* ^−/−^ cells (Fig. S1C). For BMDDCs, CXCL12 caused membrane hyperpolarization in both WT and *Gstp1/p2* ^−/− ^cells, and this hyperpolarization was stable during incubation. The effects of CXCL12 on membrane potential were slightly more pronounced in WT BMDDCs (Fig. 5C).

Next we assessed whether the different chemotactic responses of WT and *Gstp1/p2* ^−/− ^cells following CXCL12 stimulation were also reflected at other steps of intracellular signaling. As shown in Fig. 5D, treatment of BMDDCs with CXCL12 induced a rapid phosphorylation and activation of Akt and ERK in both WT and *Gstp1/p2* ^−/−^ cells. Compared to *Gstp1/p2* ^−/− ^cells, more intense phosphorylation of both Akt and ERK was observed in WT cells after CXCL12 stimulation. At 15 mins, pERK levels did drop compared to pAkt, perhaps reflecting the slightly different roles that these kinases play in regulating proliferative pathways.

To find a possible explanation for the differential chemotactic responsiveness and intracellular signaling capacity between WT and *Gstp1/p2* ^−/−^ cells, we determined chemokine CXCL12 receptor CXCR4, IP3 receptor IP3R mRNA levels as well as protein expression (Fig. 5E and F). Both WT and *Gstp1/p2* ^−/−^ BMDDCs expressed CXCR4 and IP3R, with significantly higher mRNA and protein expression levels of CXCR4 and IP3R3 in WT than in *Gstp1/p2* ^−/−^ cells, likely linked to the different redox status of those cells. In addition, existing evidence implies that expression of the thiol active phosphatase SHP-2 is important in regulating CXCR4 signaling [38], [39] and our data indicate that SHP-2 expression was lower in *Gstp1/p2* ^−/−^ than in WT cells (Fig. 5E). Furthermore, we analyzed the effect of SHP-2 specific inhibitor PHPS1 [40] and CXCR4 specific antagonist AMD3100 on CXCL12-induced migration of BMDDCs. PHPS1 or AMD3100 treatment significantly reduced the CXCL12-induced migration in both WT and *Gstp1/p2* ^−/−^ BMDDCs, although it had a greater effect on the WT cells (Fig. 5G). Taken together, the differential surface density of CXCR4 and SHP-2 in WT and *Gstp1/p2* ^−/−^ cells may correlate with the differential CXCR4 functional activity measured by the extent of CXCL12-induced cellular responses in hematopoietic cells.

## Discussion

The present studies were undertaken to determine why either genetic ablation or pharmacological inhibition of GSTP enhances myeloproliferation and migration, producing increased numbers of all committed cell lineages [15], [18]. Existing literature implies that ROS can regulate certain bone marrow niches and influence proliferative status [1]. Consequently, we undertook a series of studies to determine how various bone marrow cell types from *Gstp1/p2* ^−/−^ mice differed from their WT counterparts. Because ROS conditions have direct influence on cellular redox homeostasis, a number of thiol-dependent pathways are implicated, and these downstream targets are influenced by the presence/absence of GSTP.

Our characterization of bone marrow cells identified a number of consistent differences between WT and *Gstp1/p2* ^−/−^ cells with respect to thiol balance. For example, since GSTP can catalyze the forward reaction of S-glutathionylation [32], [41], its absence diminished the capacity of cells to establish high levels of either general or specific protein S-glutathionylation (Fig. 2 and Fig. 4C). Presumably as a consequence of this, free protein thiols and GSH levels were higher in resting BMCs, BMDDCs and Lin(−) cells from *Gstp1/p2* ^−/−^ mice (Fig. 3A and B). Moreover, in this report, we demonstrate for the first time that MALDI-MS bone imaging can be used for the simultaneous *in situ* visualization of both GSH and GSSG in sectioned bones with an intact bone marrow compartment (Fig. 3C). These results, while predominantly qualitative in nature, confirm the biochemical analyses that detail differences between GSH/GSSG in WT and *Gstp1/p2* ^−/−^ cell lineages and implicate how such differences might influence downstream events in proliferation, mobilization and/or migration of cells within the bone marrow compartment. Coincident with the S-glutathionylation differences between WT and *Gstp1/p2* ^−/−^ cells, we performed a quantitative proteome analysis using cysteine reactive ICAT labeling with Orbitrap mass spectrometry and identified other thiol active proteins, the expression levels of which were different between *Gstp1/p2* ^−/−^ and WT BMDDCs. Within this group (Table 1) were a number of redox active enzymes (aldoketo-reductase family 1, peroxiredoxin 4, prolyl 4-hydroxylase, triose-phosphate isomerase 1, transitional endoplasmic reticulum ATPase); cytoskeletal proteins (vimentin); apoptotic proteins (apoptotic chromatin condensation inducer 1); calcium binding protein (S100 calcium binding protein A9); regulatory proteins (rho GTPase-activating protein 17); scaffold proteins (ras GTPase-activating-like protein IQGAP1) and signaling proteins (serine/threonine kinase 16). Quantitative expression differences could indicate a number of other ways that GSTP genotype could influence bone marrow cell function. In particular, although their specific role in marrow cell proliferation has yet to be addressed, the relevance of S100 and kinases seems self-evident. In addition, the altered expression of IQGAP1 is interesting in light of our recent data showing that sulfiredoxin (a redox active protein with roles in deglutathionylation) has sequence homology to IQGAP proteins and involved in controlling cell migration [42], [43].

The redox status in cells is crucial in the regulation of the chemokine system, exemplified by the observations that antioxidants decrease chemokine receptor expression and chemotaxis, while H_2_O_2_ or general GSH-depletion, increases chemokine receptor expression and chemotactic responses [44]. Accordingly, while both WT and *Gstp1/p2* ^−/−^ BMDDCs expressed CXCR4, the former had significantly higher mRNA and protein expression levels (Fig. 5E and F)), implying a link to the different redox status of the cells. The thiol-dependent phosphatase SHP-2 regulates CXCR4 signaling [38], [39]. Previous studies have shown that SHP-2 functions as an adaptor molecule, which can bind to several proteins and then transduce various proliferation/migration signals. CXCR4, SHP-2 and cbl collectively participate in the formation of a multimeric signaling complex and over-expression of SHP-2 increases CXCL12-induced chemotaxis, whereas phosphatase inhibitors significantly inhibit CXCL12-induced migration [38]. The SHP-2 specific inhibitor PHPS1 or CXCR4 specific antagonist AMD3100 significantly reduced CXCL12-induced migration of WT BMDDCs, and had greater effect on the WT cells (Fig. 5G). Moreover, the phosphatase activity of SHP-2 is subject to regulation by S-glutathionylation *in vitro* [45] and the redox modifications could prove to be physiologically significant and pertinent to the observed differences between WT and *Gstp1/p2* ^−/−^ bone marrow cells (where SHP-2 expression was lower). Taken together, the differential surface density of CXCR4 and SHP-2 in WT and *Gstp1/p2* ^−/−^ cells could be correlated with the differential CXCR4 functional activity measured by the extent of chemokine-induced directional migration and the intracellular signaling capacity (e.g. PI3K, MAPKs, calcium oscillation, etc.) (Fig. S1 and Fig. 5A–D). It should be noted that CXCR4 is expressed in multiple cell types in the immune and central nervous systems, hematopoietic stem/progenitor cells, endothelial and epithelial cells and cancer cells. Its ligand, CXCL12, is expressed/secreted in various tissues and organs (bone marrow, liver, lung, skin, skeletal muscle, brain, kidney and heart). CXCL12/CXCR4 signaling plays an important and unique role in the regulation of stem/progenitor cell trafficking, inflammation, embryo/organogenesis, tissue/organ regeneration, and tumor progression, angiogenesis, metastasis, and survival [37], [46], [47]. Thus, our observed link between GSTP and different levels/activities of CXCR4 and SHP-2 is likely functional.

The calcium ionophore A23187-caused a rapid influx of extracellular Ca^2+^ into BMDDCs, a tightly regulated process that was quickly accompanied by restoration to basal levels (most likely through Ca^2+^ influx into intracellular store(s)) (Fig. 4A). However, in Lin(−) cells, the influence of A23187 on Ca^2+^ permeability and uptake is not regulated. ThG is a specific SERCA inhibitor [34] and caused a rapid increase and subsequent slow restoration of basal level of intracellular Ca^2+^ only in WT Lin(−) cells (in the absence of extracellular Ca^2+^). Treatment of BMDDCs with ThG showed similar kinetics, but with higher amplitude and a strong GSTP-dependent effect on intracellular Ca^2+^ flux (Fig. 4B). Our data show equivalent level of SERCA2 expression and S-glutathionylation in BMDDCs (Fig. 4C and D). S-glutathionylation of Cys674 (located in the cytosol-facing hinge domain) of SERCA provides a physiological, cGMP-independent mechanism of activating intracellular Ca^2+^ fluxes. Over-oxidation of this cysteine (sulfinic/sulfonic acid) prevents its S-glutathionylation and impairs the dynamics of intracellular Ca^2+^ flux [35]. The absence of GSTP in *Gstp1/p2* ^−/−^ BMDDCs results in decreased SERCA2 S-glutathionylation and consequent diminished activation (Fig. 4C). In contrast, GSTP mediates a faster increase and return of intracellular Ca^2+^ to its basal levels in BMDDCs (Fig. 4B).

Amplitude of a CXCL12-mediated oscillation of intracellular Ca^2+^ through its release from and influx into intracellular stores (E(S)R, mytochondria, etc) is proportional to GSTP level and is similar in Lin(−) cells (Fig. S1B). Both the IP_3_R channel and the plasmalemmal Ca^2+^-ATPase (PMCA) pump can be reversibly S-glutathionylated. IP3R channel activity is enhanced by glutathionylation, whereas PMCA pump activity is inhibited [48]. Our data are consistent with the hypothesis that GSTP can promote S-glutathionylation of the IP_3_R and PMCA causing Ca^2+^ release from IP_3_-sensitive internal Ca^2+^ stores and elevation of basal intracellular Ca^2+^ levels - in the absence of extracellular Ca^2+^ [49]. In Lin(−) cells, these effects cause an initial plasma membrane depolarization and GSTP-dependent additional depolarization/hyperpolarization in the presence of extracellular Ca^2+^ (Fig. S1C), most likely indicating a Ca^2+^-induced Ca^2+^-release effect. The amplitude of CXCL12 effects on mobilizing intracellular Ca^2+^ in BMDDC is also GSTP dependent and similar to that in Lin (−) cells, but with minimal oscillations in the absence of extracellular Ca^2+^ (Fig. 5B). CXCL12 can also cause a continuous, GSTP-independent hyperpolarization of plasma membranes in BMDDCs (in the presence of extracellular Ca^2+^, Fig. 5C). This conclusion is compatible with differences of plasma membrane-mediated Ca^2+^ fluxes in Lin(−) cells and BMDDCs and would fit the model where CXCL12-mediated intracellular Ca^2+^ dynamics and plasma membrane depolarization is linked with differences in GSTP mediated protein S-glutathionylation.

Overall, genetic ablation of GSTP is causally linked with multiple events that contribute to the regulation of bone marrow cell proliferation and migration. A common link is the perturbation of redox homeostasis and those documented in this paper help to explain why *Telintra*, as an inhibitor of GSTP, has clinical activity as a small molecule myeloproliferative drug [16], [17].

## Materials and Methods

### Mice

C57BL/6 wild type mice were purchased from Jackson Laboratory (Bar Harbor, ME). *Gstp1/p2* ^−/−^ mice were generated as described earlier [50]. The mice were bred and kept in the Association for Assessment and Accreditation of Laboratory Animal Care (AAALAC) - certified animal facility of the Medical University of South Carolina (MUSC). All of the mice were used at approximately 8–12 weeks of age. The Institutional Animal Care and Use Committee of Medical University of South Carolina approved all of the experimental procedures used in this study.

### Primary cells and culture conditions

The femurs and tibias were harvested from WT or *Gstp1/p2* ^−/−^ mice immediately after cervical dislocation. Crude bone marrow cells were flushed from the bones into RPMI-1640 culture medium (HyClone, Logan, UT) using 26-gauge needles and 10-ml syringes, and filtered through a 40 µm nylon cell strainer (BD, Franklin Lakes, NJ) to prepare single-cell suspensions. Red blood cells were then lysed with ACK (Ammonium-Chloride-Potassium) lysis buffer (Gibco, Life Technologies, Carlsbad, CA).

Bone marrow derived-dendritic cells were generated according to previous reported procedures [51], [52] with minor modifications. Murine BMCs (4–5×10^5^/ml, 10 ml/plate) were plated in RPMI-1640 supplemented with 10% fetal bovine serum (FBS), 100 U/ml penicillin, 100 µg/ml streptomycin (all from Mediatech, Manassas, VA), and 20 ng/ml recombinant mouse GM-CSF (BioAbChem, Ladson, SC) (DC medium) into 100-mm culture dishes (Sarstedt, Newton, NC). Fresh DC medium was added on day 4 and was gently replaced by fresh DC medium containing 10 ng/ml recombinant mouse GM-CSF on day 7. Immature BMDDCs (non-adherent and loosely adherent cells) were used in experiments on day 8.

For BM (Lin(−)) cells, BMCs were centrifuged through Lympholyte-M (Cedarlane, Burlington, NC) to isolate BM-mononuclear cells (MNCs). BM-MNCs were incubated on ice for 30 min with biotin-conjugated rat antibodies specific for murine CD4, CD8A, CD45R/B220, Gr-1 and Ter-119 (BD Pharmingen, BD Biosciences, San Jose, CA). The labeled mature lymphoid and myeloid cells were depleted by incubation on ice for 30 min with sheep anti-rat IgG Dynabeads (Invitrogen, Life Technologies, Carlsbad, CA) at a bead: cell ratio of 3∶1 with gentle rotation. Cells binding the Dynabeads were removed with a DynaMag-15 magnet (Invitrogen, Life Technologies). The negatively isolated Lin(−) cells were washed twice with PBS containing 0.1% BSA and resuspended in StemSpan™ Serum-Free Expansion Medium (StemCell technologies, Vancouver, BC, Canada) supplemented with 100 ng/ml recombinant mouse stem cell factor (Sigma, Saint Louis, MO), thrombopoietin, and Flt3L (both from BioAbChem, Ladson, SC) (Lin(−) cell medium).

### Proliferation assay

Cell proliferation was assessed using a BrdU Cell Proliferation assay kit (Cell Signaling Technology, Danvers, MA). In brief, WT or *Gstp1/p2* ^−/−^ Lin(−) cells or BMDDCs were seeded at 0.2×10^6^ cells/ml in Lin (−) cell medium or DC medium in a 96-well or 24-well plate and incubated for 48 h. BrdU solution (final concentration 10 µM) was then added and the cells kept at 37°C in the incubator for 4 h. The incorporation of BrdU into the DNA was measured by means of monoclonal anti-BrdU antibodies in a cellular ELISA format according to the manufacturer’s protocol.

### Cell treatment with H_2_O_2_ Or PABA/NO

Wild type and *Gstp1/p2* ^−/−^ BMCs or BMDDCs (1×10^6 ^cells/ml) suspended in complete medium (RPMI-1640 supplemented with 10% FBS, 100 U/ml penicillin and 100 µg/ml streptomycin) were exposed to different concentrations of H_2_O_2_ (1, 5 or 10 mM) or PABA/NO (0.5, 1 or 2 µM), at 37°C for 15 min as indicated in individual experiments. The reaction was terminated by immediate centrifugation at 500 g, 4°C for 5 min. Supernatants were removed and pellets washed once with ice-cold PBS and then solubilized by ice-cold lysis buffer [50 mM Tris-HCl (pH 7.5), 150 mM NaCl, 1% Triton, 1 mM EDTA, 1 mM EGTA, 5 mM NEM, plus a protease inhibitor cocktail (Roche Diagnostics, Indianapolis, IN)]. Cell lysates/supernatants were collected from the cells after spinning at 16,000 g for 10 min at 4°C and used for immunoblotting.

### Cell treatment with CXCL12

Wild type and *Gstp1/p2* ^−/−^ BMDDCs were harvested, resuspended at a concentration of 1×10^6^ cells/ml in RPMI-1640 and kept in the incubator at 37°C for another 2 hours to reduce the basal activity of intracellular signaling pathways. Then BMDDCs were stimulated with chemokine, 200 ng/mL CXCL12 (BioAbChem) for various times (2–15 min). Stimulation was terminated by centrifugation at 500 g, 4°C for 5 min. Supernatants were removed and pellets washed once with ice-cold PBS and then solubilized by ice-cold lysis buffer [50 mM Tris-HCl (pH 7.5), 150 mM NaCl, 1% Triton, 1 mM EDTA, 1 mM EGTA, 40 mM β glycerophosphate, 5 mM sodium pyrophosphate, 5 mM sodium fluoride, 2 mM sodium orthovandate, plus a protease inhibitor cocktail]. Cell lysates/supernatants were collected from the cells after spinning at 16,000 g for 10 min at 4°C and used for immunoblotting.

### Immunoblotting

Total soluble protein was quantitated by bicinchoninic acid (BCA) protein assay (Pierce, Rockford, IL). Cell lysates were resolved in SDS-loading buffer (80 mM Tris-HCl, pH 6.8, 2% SDS, 10% glycerol, 0.02% bromophenol blue, (±) 5 mM tris(2-carboxyethyl)phosphine (TCEP)) and heated to 95°C for 5 min. Equal amounts of protein were electrophoretically separated on 7.5%, 10% or 4–20% SDS-PAGE (BioRad, Hercules, CA) and transferred to Low Fluorescent PVDF membranes (Millipore, Billerica, MA) or nitrocellulose membranes (BioRad) by Trans-Blot Turbo Transfer System (BioRad). PVDF or nitrocellulose membranes were incubated in Odyssey blocking buffer (LI-COR, Lincoln, NE) for 1 hour to reduce non-specific binding and then probed with appropriate primary antibodies (diluted in Odyssey blocking buffer) at 4°C overnight. Immunoblots were then developed with infrared (IR) fluorescence IRDye secondary antibodies (LI-COR) at a dilution of 1∶15,000, imaged with a two-channel (red and green) IR fluorescent Odyssey CLx imaging system (LI-COR) and quantified with Image Studio 3.0 software (LI-COR). The following antibodies were used for immunoblots: rabbit polyclonal GSTP (MBL, Woburn, MA), mouse monoclonal anti-GSH (Virogen, Watertown, MA), rabbit polyclonal anti-Cdk2, mouse monoclonal anti-Phospho-ERK (Tyr204), rabbit polyclonal anti-SHP-2, mouse monoclonal anti-SERCA2 (all from Santa Cruz Biotechnology, Dallas, Texas), rabbit polyclonal anti-beta actin, rabbit polyclonal anti-CXCR4, rabbit polyclonal anti-ERK (both from Abcam, Cambridge, MA), rabbit polyclonal anti-Akt, rabbit polyclonal anti-Phospho-Akt (Ser473), rabbit monoclonal anti-IP3R (all from Cell Signaling Technology), IRDye 800CW Goat anti-Mouse IgG, IRDye 800CW Goat anti-rabbit IgG, IRDye 680RD Goat anti-mouse IgG, and IRDye 680RD Goat anti-Rabbit IgG (all from LI-COR).

### Flow cytometry

Cell surface CXCR4 expression was determined by fluorescence-activated cell sorter (FACS) analysis using FITC-conjugated rat anti-mouse CXCR4 mAb or FITC-conjugated rat IgG2b (both from BD Pharmingen) as isotype control. A total of 0.5×10^6^ cells were incubated with primary antibodies in FACS buffer (2% FBS in PBS) for 30 min at room temperature, washed once in FACS buffer, resuspended in 250 µl of FACS buffer and analyzed on a BD FACSCalibur analytical flow cytometer and CellQuest Pro software (BD Pharmingen). After gating on CD11c (APC-conjugated hamster anti-mouse CD11c mAb, Affymetrix, eBioscience, San Diego, CA) positive cells, 20,000 events per sample were analyzed.

### Immunoprecipitation of S-glutathionylated SERCA2

Five hundred micrograms of protein from WT or *Gstp1/p2* ^−/−^ BMDDCs lysates were pre-cleared by incubation with protein A/G-agarose beads (Santa Cruz Biotechnology) for 1 hour at 4°C. After the removal of protein A/G-agarose beads by brief centrifugation, the samples were incubated with mouse monoclonal anti-GSH antibody for 2 hours at 4°C. The antibody-antigen complexes were immunoprecipitated by incubating with protein A/G-agarose beads overnight at 4°C. Non-specific bound proteins were removed by washing protein A/G-agarose beads once with ice-cold lysis buffer and twice with PBS. Bound immunocomplexes were solubilized in SDS-loading buffer and analyzed by subsequent SDS-PAGE, probing of the immunoblots with goat polyclonal anti-SERCA2 antibody (Santa Cruz Biotechnology). As a reagent control, cell lysates were incubated with isotype control IgG and subjected to the same procedures.

### RNA isolation and Real-Time RT-PCR analysis

Total RNA was prepared using the Isolate II RNA mini kit (Bioline, Taunton, MA) and cDNA was then generated with the iScript cDNA synthesis kit (Bio-Rad) according to the manufacturers’ protocols. Subsequently, quantification of gene expression was performed in duplicates using iQ SYBR Green supermix (Bio-Rad) with detection on an MyiQ Real-Time PCR System (Bio-Rad). The reaction cycles used were 95°C for 5 min, and then 40 cycles at 95°C for 15 s and 58°C for 1 min followed by melt curve analysis. The following primers were used: CXCR4, forward 5′-TCAGTGGCTGACCTCCTCTT-3′, reverse 5′-CTTGGCCTTTGACTGTTGGT-3′; GAPDH, forward, 5′-CCCAGCAAGGACACTGAGCAA-3′, reverse 5′-AGGCCCCTCCTGTTATTATGG-3′; IP3R1, forward 5′-AGAAACGGAACAGGATAAG-3′, reverse 5′-AAGAAGAGGAGGTCGTAGAT-3′; IP3R2, forward 5′-CAACCAGAfCCCTGGAGAGCTTGAC-3′, reverse 5′-TTGCCCAGAGGGTTGATGTCACTC-3′; IP3R3, forward 5′-AGACCCGCTGGCCTACTATGAGAA-3′, reverse 5′-GTCAGGAACTGGCAGATGGCAGGT-3′; SERCA2, forward 5′-GATCCTCTACGTGGAACCTTTG-3′, reverse 5′-CCACAGGGAGCAGGAAGAT-3′. Relative gene expression quantification was based on the comparative threshold cycle (CT) method (2^–ΔΔCt^) with normalization of the raw data to the included housekeeping gene (beta actin).

### Measurement of intracellular reduced protein thiol levels

ThioGlo-1 (TG-1, [3H-Naphthol[2,1-b]pyran-s-carboxylic Acid, 10-(2,5-Dihydro-2,5-dioxo-1H-pyrrol-1-yl)-9-methoxy-3-oxo-, methyl ester]) (Calbiochem, San Diego, CA), a maleimide sulfhydryl-specific fluorescent probe, was used to monitor intracellular reduced protein thiols as reported earlier [31]. BMCs, Lin(−) cells or BMDDCs were harvested, washed twice with PBS and then solubilized by ice-cold lysis buffer [50 mM Tris-HCl (pH 7.5), 150 mM NaCl, 1% Triton, 1 mM EDTA, 1 mM EGTA, plus a protease inhibitor cocktail]. Cell lysates/supernatants were collected from the cells after spinning at 16,000 g for 10 min at 4°C, and low molecular weight (<6 kDa) compounds were eliminated from the cell lysates by using size-exclusion chromatography with a mirco Bio-Spin 6 column (BioRad). After measuring protein concentrations by BCA protein assay, 20 ul (∼20–50 ug) cell lysate was diluted with 2 ml 20 mM sodium phosphate buffer, pH 7.4. The total intracellular reduced protein thiol levels were then determined by an immediate fluorescence increase upon the addition of 5 µM (final concentration) of TG-1. Briefly, fluorescence was measured by a QM-4 spectrofluorometer (PTI, Birmingham, NJ) in a 10×10 mm quartz cuvette under constant stirring at 37°C, controlled by TC 125 Temperature Controller (Quantum Northwest, Shoreline, WA), with excitation (Ex) at 379 nm and emission (Em) at 513 nm using standard kinetic mode with resolution of 0.1 sec. The emission of each sample was recorded for 100 seconds (background) before and until 500 seconds after the addition of TG-1. Saturated TG-1 fluorescence values were corrected for background emissions and normalized for protein content, and averaged using SigmaPlot 10.0 software (Systat Software, San Jose, CA).

### Measurement of GSH and GSSG levels

BMCs, Lin(−) cells or BMDDCs were harvested and cell lysates were prepared in the same way as described above. Reduced GSH was determined by a fluorometric method (Ex = 379 nm, Em = 513 nm), using Cayman Thiol Detection Assay Kits (Ann Arbor, Michigan) according to the manufacturer’s protocol with few modifications. Proteins were precipitated from cell lysates by adding 5% trichloroacetic acid (Sigma-Aldrich, St. Louis, MO). After 10 min at −20°C, supernatants were collected after spinning at 16,000 g for 10 min at 4°C. GSH standards (0.0625 to 5 µM) were prepared by dilution in dH_2_O. The concentrations of GSH in cell lysates are expressed in nmol/mg protein. The determination of oxidized GSSG is based on its reduction by glutathione reductase using NADPH as a source of electrons and on measurement of NADPH fluorescence decrease at 37°C. The final concentrations of reagents were 2.66 µg/ml glutathione reductase and 50 µM NADPH in 1.5 ml 50 mM Tris-HCl pH 8.0 containing 1 mM EDTA and 10 µM sodium azide. The enzymatic reaction was monitored for 50 seconds before and until 300 seconds after the addition of each sample or GSSG standard with excitation at 340 nm and emission at 460 nm (resolution 0.1 sec). The concentrations of GSSG in cell lysates are expressed in nmol/mg protein. In both cases, the fluorescence was recorded by a QM-4 spectrofluorometer in standard kinetic mode as described above. GSH, GSSG, NADPH and glutathione reductase were all purchased from Sigma-Aldrich (St. Louis, MO).

### ICAT labeling and nanohplc-Orbitrap Elite mass spectrometry

BMDDCs were harvested and the pellet was dissolved in ICAT denaturation buffer [100 mM Tris-HCl (pH 8.5), 4 M urea, 0.05% SDS, plus a protease inhibitor cocktail]. Protein (100 ug) was treated with 1.25 mM TCEP to reduce the cysteine residues and labeled with cleavable, cysteine reactive ICAT reagents according to the manufacturer’s instructions (AbSciex, Foster city, CA). Protein from WT and *Gstp1/p2* ^−/−^ mouse was labeled with ‘light (L)’ and ‘heavy (H)’ ICAT reagents, respectively. ICAT labeling and nano-HPLC-Orbitrap Elite MS were performed at the Proteomics Core Facility of the Medical University of South Carolina. Protein identification was performed using Mascot and Sequest nodes within the Proteome Discoverer 1.3 platform (Thermo Scientific) against a RefSeq murine database (28925 entries, downloaded 03/05/12). Parameters for peptide identification were as follows: precursor mass tolerance of 100 ppm, fragment mass tolerance of 0.8 Da, one missed cleavage, dynamic modifications of cysteine with light or heavy labeled ICAT reagent, deamidation of asparagine and glutamine, and methionine oxidation. Only high confidence peptides with a false discovery rate (FDR) <1% were included. The ICAT quantitation was performed using Proteome Discoverer 1.3 by calculating the relative intensities of the light and heavy ICAT labeled pairs. All ratios were normalized using the median ratio from all peptides identified.

### MALDI-MS bone imaging

The femurs of mice were harvested and snap frozen in the vapor phase of liquid nitrogen for at least 10 min, and then stored at –80°C until analysis. A cryostat (Microm HM550, Thermo) was used to slice the frozen bones to a thickness of 10 µm. The bone slice was attached to double sided carbon tape (SPI supplies, West Chester, PA), then mounted onto a conductive indium tin oxide coated (ITO) slide (Bruker Daltonics, Billerica, MA). Slides were desiccated at room temperature for at least 15 min before matrix application. MALDI matrix (9-aminoacridine, 5 mg/ml in 70% ethanol) was applied to the bone slides using an ImagePrep spray station (Bruker Daltonics).

MALDI-MS analysis was performed using a Solarix 7T dual source ESI/MALDI Fourier transform ion cyclotron resonance (FTICR) mass spectrometer (Bruker Daltonics). Acquisition was set up using FlexControl 3.0 and FlexImaging 4.0 software (Bruker Daltonics). Glutathione metabolites were detected in negative ion mode analyzing the mass range m/z = 50–800 with a SmartBeam II laser operating at 1000 Hz, a laser spot size of 25 µm, and a raster width of 200 µm. For each laser spot, 500 spectra were averaged and all data was normalized using root means square. Images were generated using FlexImaging 4.0 software (Bruker Daltonics). Structural confirmation of GSH and GSSG was done by collision-induced fragmentation of the indicated ions.

### Chemotaxis assay

Chemotaxis assays were performed in 24-well transwell chambers with polycarbonate membranes (5 µm pore size; Corning, NY) as described by Branham-O’Connor et al [52]. In brief, 600 µl of either chemotaxis buffer (RPMI-1640 with 200 ng/ml CXCL12) or as a control RPMI-1640 alone were placed in the lower chambers. Upper chambers were loaded with 100 µl cell suspensions of Lin(−) cells or BMDDCs at a concentration of 3×10^6^ cell/ml in RPMI 1640. Where indicated, BMDDCs were preincubated with 10 µM PHPS1 or 1 µM AMD3100 (both from Calbiochem) in serum-free RPMI for 2 h at 37°C prior to measuring chemotaxis. The complete chamber was kept at 37°C in the incubator for 6 or 16 h. After that, cells remaining in the upper chambers and cells that had migrated through the membrane to the lower chambers were counted with a Z1 Coulter Particle Counter (Beckman Coulter, Hialeah, FL). The percentage of migration was determined as migrated/total cells. To calculate the percentage of specific migration induced by chemokine CXCL12, the percentage of cells migrating to medium alone (control) was subtracted from the percentage of cells migrating to medium with CXCL12.

### Intracellular calcium oscillations

Lin(−) cells or BMDDCs were harvested, re-suspended at a concentration of 1×10^6^ cell/ml and incubated with 5 µM Fluo-3-AM (Invitrogen, Life Technologies, Carlsbad, CA) at 37°C for 45 min in RPMI-1640 at 37°C in the dark. All subsequent manipulations were performed with the Fluo-3-AM labeled cells protected from light. The cells were washed three times with PBS to remove extracellular dye and then re-suspended at a concentration of 1×10^6^ cell/ml in PBS, containing 100 µM CaCl_2_ (Ca^2+^-extracellular solution) or 2 mM EGTA (zero Ca^2+^-extracellular solution) immediately prior to use. The kinetics of intracellular free ionized calcium ([Ca^2+^]_i_) changes were measured using a QM-4 spectrofluorometer and standard kinetic mode with excitation at 506 nm and emission at 526 nm at 37°C under constant stirring. The emission of each sample was recorded for ∼150 seconds before and until 500 seconds after the addition of 1 µM A23187 (0.1% DMSO); 1 µM ThG (0.1% DMSO) (both from Sigma-Aldrich, St. Louis, MO); 100 ng/ml CXCL12. A23187 (1 mM) and ThG (1 mM) stock solutions were prepared in DMSO, while CXCL12 (100 µg/ml) was reconstituted in PBS containing 0.1% BSA. All fluorescent measurements were corrected for fluorescence of unlabeled cells. Representative traces of three independent experiments were averaged and smoothed using standard Sigma-Plot 10.0 software (Systat Software, San Jose, CA).

### Plasma membrane potential measurements

Plasma membrane potential was determined using the slow-response potential-sensitive dye DiSBAC2(3) (Invitrogen, Life Technologies, Carlsbad, CA). Lin(−) cells or BMDDCs were harvested, washed three times with PBS, and re-suspended at a concentration of 0.5×10^6^ cell/ml in PBS with 100 µM CaCl_2_. Cell suspensions were incubated with 5 µM (final concentration) DiSBAC2(3) in a quartz cuvette under constant stirring at 37°C for 3 min in the dark. Then the kinetics of the emission at 560 nm (Ex = 530 nm) were monitored by a QM-4 spectrofluorometer. The emission of each sample was recorded for 100 seconds before and until 500 seconds after the addition of 100 ng/ml CXCL12. Representative traces of three independent experiments were averaged and smoothed using standard Sigma-Plot 10.0 software (Systat Software, San Jose, CA).

### Statistical analysis

Student’s *t* tests were used where *P* values<0.05 were regarded as statistically significant. Data were expressed as means ± *SD* with *n* equal to the number of animals/group examined under each condition.

## Supporting Information

Figure S1 **Lin(**−**) cell responses to CXCL12.** (*A)* Chemotaxis of Lin(−) cells to CXCL12. Wild type and *Gstp1/p2* ^−/−^ Lin(−) cells were either untreated (control) or stimulated with 200 ng/ml CXCL12 for 6 h. Values are average percentages of migration (±*SD*) from three independent experiments, with asterisks (*) indicating statistical significant differences between WT and *Gstp1/p2* ^−/−^ Lin(−) cells (*p*<0.05). To calculate the specific chemotaxis induced by CXCL12, the percentage of cells migrating to medium alone (control) was subtracted from the percentage of cells migrating to medium with CXCL12. *(B, C*) Intracellular calcium *(B)* and plasma membrane potential *(C)* dynamics in WT and *Gstp1/p2* ^−/−^ Lin(−) cells in response to CXCL12. The arrows indicate the addition of CXCL12. Data are representative traces of three independent experiments.
(TIF)
