# Correction: Glutathione S-Transferase P Influences Redox and Migration Pathways in Bone Marrow

**DOI:** 10.1371/journal.pone.0119266

**Published:** 2015-03-05

**Authors:** The PLOS ONE Staff

There is an error in the last sentence of the Abstract. The correct sentence is: These observed differences contribute to our understanding of how genetic ablation of GSTP causes different levels of myeloproliferation and migration.

There is an error in Fig. 3A. Please see the corrected Fig. 3 here.

**Fig 3:**
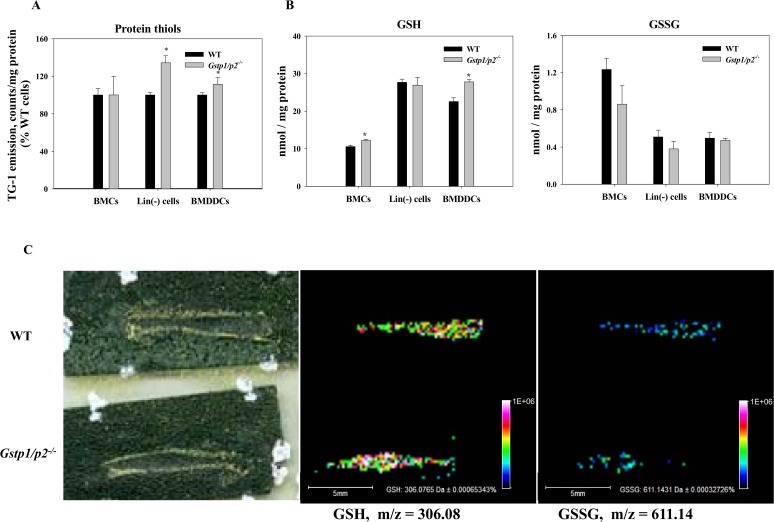
Protein thiols, GSH and GSSG levles in bone marrow cells. (*A, B*) Intracellular reduced protein thiols (A), and GSH/GSSG levels (B) in crude BMCs, Lin(−) cells and BMDDCs. Intracellular reduced thiol and GSH levels were measured by means of a sulfhydryl-specific fluorescent probe; intracellular GSSG levels were determined based on the reduction of GSSG in the presence of glutathione reductase and NADPH and on measurement of NADPH fluorescence decrease. Values are means (±*SD*) from at least three independent experiments, with asterisks (*) indicating statistical significant differences between (*p*<0.05). (*C*) Representative MALDI-MS images of GSH and GSSG in sectioned femur showing bone marrow distribution in WT and *Gstp1/p2^−/−^* mice. From left to right: scanned image of matrix sprayed MALDI slide of mouse femur with bone marrow; corresponding images of: GSH ions at m/z = 306.08 and GSSG ions at m/z = 611.14. Color heat map of the data points in the GSH and GSSG images represent averaged individual ion signal intensities of the spots.

The last sentence of the subsection “Increased DNA synthesis in *Gstp1/p2^−/−^* bone marrow cell populations” of the Results should have cited references 15 and 18 instead of 26. The correct sentence should read: These results support our previous publication that ablation of GSTP either genetically or pharmacologically results in the over-production of lymphoid, erythroid and myeloid lineage cell lineages as well as platelets [15, 18].
